# Cold acclimation affects immune composition in skeletal muscle of healthy lean subjects

**DOI:** 10.14814/phy2.12394

**Published:** 2015-07-06

**Authors:** Anouk A J J van der Lans, Mariëtte R Boon, Mariëlle C Haks, Edwin Quinten, Gert Schaart, Tom H Ottenhoff, Wouter D van Marken Lichtenbelt

**Affiliations:** 1 Department of Human Biology, NUTRIM, School for Nutrition, Toxicology and Metabolism, Maastricht UniversityMaastricht, The Netherlands; 2 Department of Medicine, Division of Endocrinology, Leiden University Medical CenterLeiden, The Netherlands; 3 Department of Infectious Diseases, Leiden University Medical CenterLeiden, The Netherlands; 4 Department of Human Movement Sciences, NUTRIM, School for Nutrition, Toxicology and Metabolism, Maastricht UniversityMaastricht, The Netherlands

**Keywords:** Cold acclimation, macrophages, skeletal muscle, Th17 cells, thermogenesis

## Abstract

Low environmental temperatures have a profound effect on biological processes in the body, including the immune system. Cold exposure coincides with hormonal changes, which may directly or indirectly alter the immune system, even in the skeletal muscle. The aim of the present study was to investigate the effect of cold acclimation on immune composition in skeletal muscle. Skeletal muscle biopsies were obtained from 17 healthy lean subjects before and after 10 days of mild cold exposure (15**°**C, 6 h/day). Nonshivering thermogenesis was calculated by indirect calorimetry. We found that cold acclimation increased nonshivering thermogenesis from 10.8 ± 7.5 before to 17.8 ± 11.1% after cold acclimation (*P* < 0.01), but did not affect plasma catecholamine nor cytokine levels. In contrast, cold acclimation affected mRNA expression of several immune cell markers in skeletal muscle. It downregulated expression of the Th17 markers RORC (−28%, *P* < 0.01) and NEDD4L (−15%, *P* < 0.05), as well as the regulatory T-cell marker FOXP3 (−13%, *P* < 0.05). Furthermore, cold acclimation downregulated expression of the M2 macrophage markers CCL22 (−50%, *P* < 0.05), CXCL13 (−17%, *P* < 0.05) and CD209 (−15%, *P* < 0.05), while the M1 macrophage marker IL12B was upregulated (+141%, *P* < 0.05). Cold acclimation also enhanced several markers related to interferon (IFN) signaling, including TAP1 (+12%, *P* < 0.01), IFITM1/3 (+11%, *P* < 0.05), CD274 (+36%, *P* < 0.05) and STAT 2 (+10%, *P* < 0.05). In conclusion, 10 days of intermittent cold exposure induces marked changes in the expression of immune cell markers in skeletal muscle of healthy lean subjects. The physiological consequences and therapeutic relevance of these changes remain to be determined.

## Introduction

During cold exposure, the body attempts to maintain a constant internal body temperature by increasing heat production and minimizing heat loss, both of which are mediated by activation of the sympathetic nervous system (van Marken Lichtenbelt and Schrauwen 2011). The increase in heat production is accomplished through shivering thermogenesis (e.g., involuntary muscle contractions) as well as nonshivering thermogenesis (NST) in which both muscle and brown adipose tissue are involved (Cannon and Nedergaard 2004).

Low environmental temperatures have a profound effect on biological processes in the body, including the immune system. To date, only few studies have investigated the effect of acute (<4 h) cold exposure on plasma immune cell composition and cytokine production in humans. Immersion of healthy young men in cold (14°C) water or exposure to air (5°C) induced a rapid increase in neutrophils (Brazaitis et al. 2014), B-cells, and T-cells as well as Natural Killer (NK) cell activity in the blood (Brenner et al. 1999), while monocyte numbers had decreased (Brazaitis et al. 2014), suggesting both pro- and anti-inflammatory effects of acute cold exposure. On the other hand, a recent study by Kox et al. (Kox et al. 2014) showed that acute activation of the sympathetic nervous system by, amongst others, acute cold attenuated the pro-inflammatory innate immune response upon LPS injection in humans, supporting a merely anti-inflammatory effect of acute cold exposure.

Several mechanisms may underlie the link between cold exposure and the immune system. Cold exposure coincides with hormonal changes, like catecholamine release, which either directly or indirectly may alter immune status (Brenner et al. 1999; Brazaitis et al. 2014; Gagnon et al. 2014). Indeed, several immune cell types including Natural Killer cell (NK cells), B and T cells, contain *β*-adrenergic receptors that are targeted by catecholamines to induce a cascade of events resulting in immune cell mobilization (Brenner et al. 1999). Furthermore, immune cells contain cold sensing transient receptor potential channels that affect intracellular signaling upon activation (Xiao et al. 2011) and this may provide an additional mechanistic link between cold exposure and immune status. Besides plasma catecholamines, acute cold exposure also alters levels of several other hormones and cytokines in the plasma, including cortisol and interleukin-6 (Brenner et al. 1999; Kox et al. 2014).

As mentioned previously, skeletal muscle is involved in shivering and nonshivering thermogenesis. Since immune cells, and in particular macrophages, serve an important role in skeletal muscle integrity (Arnold et al. 2007) and cold exposure affects immune cells in plasma, we hypothesized that cold acclimation affects immune composition in skeletal muscle. Moreover, we anticipated that this may be mediated by catecholamines as well as other hormones and cytokines that are released upon cold exposure.

To this end, we studied immune composition in skeletal muscle biopsies of 17 healthy lean subjects that were subjected to longer term (10 days) mild cold exposure (15**°**C, 6 h/day) (van der Lans et al. 2013). Before and after cold acclimation, we investigated plasma catecholamine and cytokine levels and expression of several immune cell markers, including markers for macrophages, NK cells, T cells, and pattern recognition receptors, in skeletal muscle biopsies.

## Materials and Methods

### Subjects

Seventeen healthy lean (BMI <25 kg/m^2^) subjects, of which nine females and eight males (age: 23 ± 2.3 years) participated in this study (van der Lans et al. 2013). Exclusion criteria were diabetes mellitus, pregnancy, physical activity more than twice a week, use of beta-blockers and a history of cardiovascular diseases and asthma or other pulmonary obstructive diseases. The ethics committee of Maastricht University Medical Centre+ approved the protocol, and all subjects provided written informed consent. All procedures were conducted according to the principles of the Declaration of Helsinki.

### Study design

Cold acclimation was achieved by exposure of subjects to 15–16°C for 10 consecutive days: 2 h on the first day, 4 h on the second day and 6 h per day for the remaining 8 days. Before and after this period, muscle biopsies were taken. On a separate day, body composition was determined by means of dual x-ray absorptiometry (type A; Hologic, Bedford, MA), which was followed by an individualized cooling protocol during which energy expenditure (EE) was continuously assessed (indirect calorimetry) and venous blood samples were taken.

### Cold acclimation

During the cold acclimation period, subjects were placed in a cold room that was air-cooled to an environmental temperature of 15–16°C. Subjects wore shorts and T-shirts. During the cold acclimation period, the participants were instructed to refrain from physical activity and performed sedentary activities, such as studying and watching TV.

### Skeletal muscle biopsy procedure

Before and after the cold acclimation period, muscle biopsies from the *m. vastus lateralis* (approximately 75–100 mg) were collected in fasted condition under localized anesthesia, according to the technique of Bergström (Bergstrom et al. 1967). Muscle samples were directly frozen in melting isopentane and stored at −80°C until further analysis.

### Individualized cooling protocol and indirect calorimetry

To assess the effect of cold acclimation on heat production, during an individualized cooling protocol EE was measured continuously by means of a ventilated hood system (Omnical; Jaeger, Den Haag, the Netherlands). To this end, subjects were wrapped in a water-perfused suit (ThermaWrap Universal 3166 MTRE; Advanced Technologies Ltd., Feasterville-Trevose, PA) connected to two water heating/cooling systems (Blanketroll III; SubZero, Cincinnati, OH). The protocol started with a thermoneutral period of 45 min, followed by the individualized cooling protocol (van der Lans et al. 2014). In short, each subject was cooled down until shivering occurred. After this, subjects were warmed up for several minutes so that shivering disappeared and finally suit temperature was set slightly above the temperature at which shivering started for another 30 min. NST was calculated as the difference in EE between thermoneutrality and stable mild cold exposure (last 30 min of the cooling protocol).

### Laboratory analysis

During the individualized cooling protocol, blood was collected at thermoneutrality and during mild cold exposure. Plasma concentrations of glucose (ABX Glucose HK CP, Radiometer, Horiba ABX, Montpellier, France), free glycerol (Glycerol kit; R-Biopharm, Darmstadt, Germany), and total glycerol (ABX Triglycerides CP, Radiometer, Horiba ABX) were determined on a COBAS FARA centrifugal spectrophotometer (Roche Diagnostics, Woerden, the Netherlands). Triglyceride levels were calculated using the difference in total and free glycerol. Plasma catecholamines were determined using reagents from Recipe (Recipe Chemicals and Instruments, München, Germany) and analyzed on a HPLC and by electrochemical detection. Serum insulin was analyzed with a Human Insulin-Specific RIA Kit (Millipore) on a Gamma Counter (2470 Automatic Gamma Counter Wizard; Wallac, PerkinElmer, Waltham, MA). The plasma inflammatory marker C-reactive protein (CRP) was measured with a particle-enhanced immunoturbidimetric assay on a COBAS c311 system (Roche Diagnostics GmbH, Mannheim, Germany), and IL-6 and IL-8 were measured with a chemiluminescent immunometric assay on an IMMULITE 1000 system (Siemens, München, Germany).

### RNA isolation

Total RNA was isolated from skeletal muscle biopsies (approximately 25–30 mg) using the phenol-chloroform extraction method (Tripure RNA Isolation reagent, Roche, Mannheim, Germany) and treated with a DNAse kit (TURBO DNAse, Life Technologies, Breda, the Netherlands) according to the manufacturer’s instruction. Amount of RNA was determined by NanoDrop.

### dcRT-MLPA assay

A dual-color reverse transcriptase multiplex ligation-dependent probe amplification (dcRT-MLPA) assay was performed as described previously (Joosten et al. 2012). Briefly, for each target-specific sequence, a specific RT primer was designed located immediately downstream of the left and right hand half-probe target sequence. Following reverse transcription, left and right hand half-probes were hybridized to the cDNA at 60°C overnight. Annealed half-probes were ligated and subsequently amplified by PCR (33 cycles of 30 sec at 95°C, 30 sec at 58°C, and 60 sec at 72°C, followed by 1 cycle of 20 min at 72°C). PCR amplification products were 1:10 diluted in HiDi formamide-containing 400HD ROX size standard and analyzed on an Applied Biosystems 3730 capillary sequencer in GeneScan mode (Applied Biosystems, Paisley, UK).

Trace data were analyzed using the GeneMapper software package (Applied Biosystems). The areas of each assigned peak (in arbitrary units) were exported for further analysis in Microsoft Excel spreadsheet software. Data were normalized to beta-2-microglobulin and signals below the threshold value for noise cutoff in GeneMapper (log2 transformed peak area 7.64) were assigned the threshold value for noise cutoff.

### Immunohistochemical stainings

To examine if any putative changes in mRNA expression levels of macrophage markers originated from invasion of muscle tissue by macrophages, we performed immunofluorescence staining in muscle cross sections. In brief, 7 μm thick cryosections were thaw-mounted on glass slides and incubated (1:100) at room temperature with a monoclonal mouse antibody directed against human CD68 (M0178, Clone EBM11, DAKO, Eindhoven, the Netherlands), a generic macrophage marker protein. The primary antibody was visualized with an anti-mouseAlexaFluor488-conjugated secondary antibody. Nuclei were stained with DAPI (4′,6-diamidino-2-phenylindole). Images were captured with a Nikon E800 fluorescence microsocope.

### Statistics

Statistical analyses were performed with PASW Statistics 20.0 for Mac (SPSS Inc., Chicago, IL). Two-sided paired sample t tests were used to compare findings between thermoneutral and mild cold conditions and to test the acclimation effects. *P* < 0.05 was considered statistically significant.

## Results

### Clinical characteristics

Of the 17 subjects who participated in the study, nine were female and eight were male. Mean age was 23 ± 2.3 years, mean BMI was 21.6 ± 2.2 kg/m^2^ and mean fat mass 22.3 ± 8.1% (Table1). Anthropometric values did not change upon cold acclimation (van der Lans et al. 2013).

**Table 1:** Subject characteristics.

Characteristic	Group (*n* = 17)
Ratio males/females	8M/9F
Age (year)	23 ± 3.2
Body mass (kg)	68.4 ± 11.7
Height (m)	1.78 ± 0.09
BMI (kg/m^2^)	21.6 ± 2.2
Body fat (%)	22.3 ± 8.1

Values are expressed as means ± SD.

### Cold acclimation and energy expenditure

Cold acclimation did not affect resting metabolic rate (RMR) (Table2). However, cold acclimation increased NST from 10.8 ± 7.5% before to 17.8 ± 11.1% after cold acclimation (*P* < 0.01) (Table2). There was no difference between males and females, both before and after the cold acclimation (data not shown).

**Table 2:** Energy expenditure and blood parameters under thermoneutral condition and during mild cold exposure, before and after cold acclimation.

Variable	Before	After
Energy expenditure (kJ/min)		
Thermoneutral	4.7 ± 0.7	4.7 ± 0.7
Mild cold	5.3 ± 0.8^C^	5.5 ± 0.8^C^
RMR (MJ/24 h)	6.8 ± 1.0	6.8 ± 1.0
Nonshivering thermogenesis (NST; %)	10.8 ± 7.5	17.8 ± 11.1^A^
Insulin (*μ*U/mL)	9.8 ± 3.9	8.8 ± 3.7
Glucose (mmol/L)	4.8 ± 0.4	4.8 ± 0.4
HOMA-index	2.1 ± 1.0	1.8 ± 0.8
Free fatty acids (*μ*mol/L)	619 ± 248	584 ± 296
Triglycerides (*μ*mol/L)	656 ± 327	530 ± 190
Noradrenalin (ng/L)		
Thermoneutral	332.6 ± 123.6	355.4 ± 127.2
Mild cold	867.5 ± 237.4^C^	916.7 ± 282.7^C^
Change upon cold stimulation	528.6 ± 261.5	578.5 ± 274.4
Adrenalin (ng/L)		
Thermoneutral	41.7 ± 17.8	33.7 ± 16.9^A^
Mild cold	34.6 ± 11.8	39.9 ± 18.6^D^
Change upon cold stimulation	−9.1 ± 20.5	5.5 ± 15.2^D^
CRP	<1	<1
IL-6	<2	<2
IL-8	<5	<5

Results are expressed as mean ± SD.

A *P* < 0.01 before versus after

B *P* < 0.05 before versus after

C *P* < 0.01 thermoneutral versus mild cold

D *P* < 0.05 thermoneutral versus mild cold. The effect of cold acclimation was tested with a paired samples t test.

### Cold acclimation and metabolic parameters

Thermoneutral plasma values of insulin, glucose, free fatty acids, and triglycerides did not change after cold acclimation (Table2). The individualized cooling protocol significantly increased plasma noradrenalin levels to the same extent both before and after cold acclimation, as previously described (Orava et al. 2011; Vosselman et al. 2012). The cooling protocol did not change plasma adrenalin levels. However, the cold acclimation period caused significantly lower adrenalin levels during thermoneutral condition (before: 41.7 ± 17.8 ng/L, after: 33.7 ± 16.9 ng/L, *P* < 0.05) and higher levels during mild cold stimulation (before: 34.6 ± 11.8 ng/L, after: 39.9 ± 18.6 ng/L, *P* < 0.01). Again, the response between males and females concerning the above-mentioned plasma values was not significantly different (data not shown). Therefore, we decided to pool the data for males and females in the following section.

### Cold acclimation and markers of systemic and muscle inflammation

We assessed the thermoneutral plasma inflammatory factors CRP, IL-6, and IL-8 before and after the cold acclimation period, but these were below their respective detection limits (Table2).

**Figure 1:**
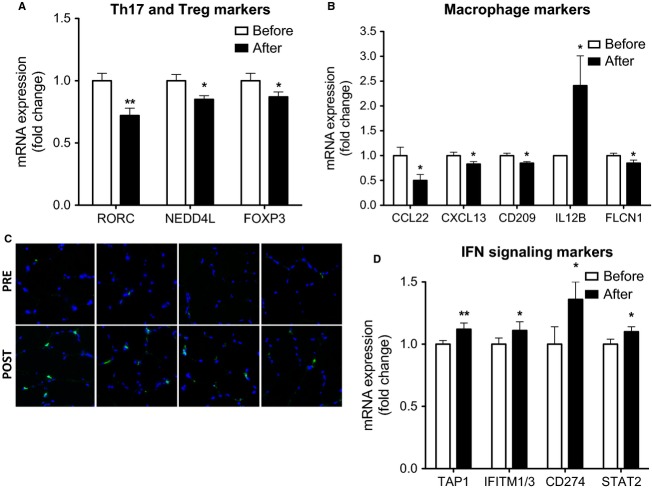
Expression of adaptive and innate immune markers in muscle biopsies before and after 10 days of cold acclimation in healthy subjects. mRNA expression levels of genes related to Th17 and Treg signaling (A), macrophage markers (B), and interferon signaling (D) were measured in skeletal muscle biopsies of healthy subjects (*n* = 17) obtained before (white bars) and after (black bars) 10 days of cold acclimation. Expression levels are normalized to the housekeeping gene B2 microglobulin and expressed as fold change compared to baseline as mean ± SEM. (C) Immunofluorescence staining of CD68 in skeletal muscle cross sections. CD68 positive macrophages are stained in green, nuclei are stained in blue. *P < 0.05, **P < 0.01, and ***P < 0.001 versus before cold acclimation.

Using the dcRT-MLPA assay, we measured mRNA expression of a large panel of inflammatory genes, including markers for innate and adaptive immune cells, pattern recognition receptors, and cytokines in muscle biopsies before and after the cold acclimation (see Suppl Table1 for the complete list). Cold acclimation resulted in a significantly lower expression of RORC (−28%, *P* < 0.01) and NEDD4L (−15%, *P* < 0.05), both involved in Th17 response (Fig.1A). In addition, the expression of the regulatory T-cell marker FOXP3 was downregulated (−13%, *P* < 0.05).

Furthermore, cold acclimation affected several markers involved in innate immune response (Fig.1B). More specifically, we found a downregulation of the M2 macrophage markers CCL22 (−50%, *P* < 0.05), CXCL13 (−17%, *P* < 0.05) and CD209 (−15%, *P* < 0.05). In contrast, the M1 macrophage marker IL12B was upregulated (+141%, *P* < 0.05). FLCN1, a marker involved in tumor-associated macrophages, was downregulated upon cold acclimation (−15%, *P* < 0.05). To investigate whether the changed expression of macrophage markers was accompanied by a change in recruitment of macrophages, we stained muscle biopsies for CD68 by immunofluorescence. Interestingly, the cold acclimation enhanced the amount of CD68-expressing cells pointing to infiltration of macrophages (stained in green, see Fig1C for four representative subjects).

Of note, cold acclimation also enhanced several markers related to interferon (IFN) signaling, including TAP1 (+12%, *P* < 0.01), IFITM1/3 (+11%, *P* < 0.05), CD274 (+36%, *P* < 0.05), and STAT 2 (+10%, *P* < 0.05) (Fig1D).

## Discussion

Several studies have shown that short-term cold exposure affects the immune system in humans, an effect that may be mediated via sympathetic activation as well as by factors released by skeletal muscle. Up to date, no studies have investigated the effect of longer term cold acclimation on immune cell composition in skeletal muscle. In the present study we demonstrate that cold acclimation (10 days, 6 h/day) affects the expression of several immune markers in skeletal muscle, in parallel with a significant increase in whole body NST. While markers involved in Th17 response and M2 macrophage markers were downregulated, markers for M1 macrophages and Th1 immunity were upregulated. This was accompanied by an increase in the number of local CD68-positive cells. Furthermore, immune markers related to IFN signaling were upregulated. Thus, cold acclimation markedly affects immune composition in skeletal muscle.

To the best of our knowledge, this is the first report describing the effect of a cold acclimation protocol on immune markers in skeletal muscle in humans. We have previously shown that markers of immune subsets in skeletal muscle can be markedly affected by dietary stimuli (Boon et al. 2015), as 5 days of high-fat high calorie diet upregulated several genes related to M1 macrophages. Interestingly, markers of M1 macrophages were also upregulated in the current study, while M2 macrophage markers were downregulated, suggesting that both high-fat diet feeding and cold elicit pro-inflammatory effects on macrophages. Moreover, we found increased CD68+ cells in skeletal muscle biopsies, suggesting influx of macrophages. This is in line with reports that show that acute cold exposure reduces circulating monocytes (Brazaitis et al. 2014), which may thus be the consequence of extravasation of monocytes from the plasma and differentiation into macrophages in peripheral tissues, including skeletal muscle. Indeed, acute cold exposure also enhances MCP-1 levels, the attraction factor for monocytes, in plasma (Brazaitis et al. 2014). As macrophages are the first line of defense against many pathogens and tissue damage, the increase in M1 macrophages in muscle may thus point to an increased preparedness of the body against possible danger. Indeed, pro-inflammatory M1 macrophages are observed experimentally in the context of muscle repair, and are usually found at early stages after muscle injury (Arnold et al. 2007). Furthermore, M1 macrophages are thought to be involved in the development of muscle insulin resistance as pro-inflammatory cytokines including TNF-*α* hamper insulin signaling in skeletal muscle (Varma et al. 2009).

An interesting finding of the current study is the decrease in markers of Th17 signaling in skeletal muscle. Th17 cells are a subtype of T helper cells that have been shown to be present in skeletal muscle (Bettelli et al. 2007; Fasth et al. 2009) and are potent inducers of tissue inflammation (Korn et al. 2009). Furthermore, they are associated with the pathogenesis of several autoimmune diseases in humans, including rheumatoid arthritis (Kirkham et al. 2006), inflammatory bowel disease (Duerr et al. 2006) and psoriasis (Krueger et al. 2007). Thus, the reduction in markers of Th17 signaling upon cold acclimation may point to a beneficial effect on autoimmunity. This is also suggested by the recent paper of Kox et al. (Kox et al. 2014), who showed that acute activation of the sympathetic nervous system by, amongst others, acute cold attenuated the pro-inflammatory innate immune response upon LPS injection in humans. More specifically, this translated in lower release of TNF-*α*, an important pro-inflammatory mediator. On the other hand, Th17 cells play an important role in orchestrating host defense against fungal infections and patients that exhibit a defect in Th17 function suffer from persistent fungal infections (Eyerich et al. 2008). So, balanced Th17 responses are of primary importance.

Several studies suggest that cold exposure is associated with an increased risk of viral infections (Chen et al. 1993; Ben-Nathan et al. 1996; Shephard and Shek 1998). An important mediator in viral infections are interferons. In this respect, our results showing an increase in interferon-inducible genes in skeletal muscle biopsies are particularly interesting as this suggests that cold acclimation enhances preparedness against viral infections. This finding is supported by previous literature showing that cold exposure enhances activity of NK cells in plasma, an important source of interferons (Brenner et al. 1999). Thus, it may be questioned whether cold exposure is indeed disadvantageous for the immune system with respect to susceptibility for viral infections.

Alterations in sympathetic nervous system activity likely contribute to the link between cold exposure and changes in the immune system. When activated by cold stress, the adrenal medulla secretes catecholamines (e.g., adrenalin and noradrenalin) into the blood and catecholamines are known to mobilize leukocytes from several pools (Brenner et al. 1999). More specifically, several immune cell types, including T and B cells and NK cells, exhibit *β* adrenergic receptors of which activation result in mobilization of these cell types. Previous studies have shown that short-term cold exposure enhances catecholamine release (Brenner et al. 1999; Gagnon et al. 2014; Kox et al. 2014). Also in the current study, we found that the personalized cooling protocol enhanced noradrenalin release in the plasma. However, we could not find differences in catecholamine levels upon cold acclimation. This may be due to the fact that the blood samples were taken the day after the last acclimation day, that is, perhaps catecholamine levels were already normalized. The changes we found on immune markers in skeletal muscle are thus likely the result of repeatedly enhanced catecholamine levels that occurred during the acclimation periods. Interestingly, cold acclimation did cause significantly lower adrenalin levels during thermoneutral condition and higher levels during mild cold stimulation. This is in line with the trend found by Vybíral et al. (Vybiral et al. 2000), who found that winter swimmers have nonsignificant (trend) lower adrenalin levels during cold exposure when compared with control subjects. A possible explanation might be an increased sensibility of the adrenergic receptor.

A potential limitation of the current study could be that we determined immune cell markers via MLPA assay without performing flow cytometry analyses, which would have allowed the integration and correlation of RNA expression data with changes in immune cell subsets composition in muscle tissue. However, performing flow cytometry analyses would require relatively large amounts of muscle tissues, the collection of which was not feasible in the current study. In addition, genetic expression assays more readily allow the assessment of differences in the quantitative expression levels of markers compared to cell surface staining techniques. Indeed, we found that, per immune group, several markers were consistently up- or downregulated, suggesting that our data are a reliable reflection of the effect of cold acclimation on immune cell composition in skeletal muscle.

In conclusion, the present study demonstrates that 10 days of cold acclimation not only results in enhanced nonshivering thermogenesis, but also induces marked changes in immune cell markers in skeletal muscle of healthy lean subjects. Markers involved in Th17 response and M2 macrophage markers were downregulated, and markers for M1 macrophages were upregulated. Furthermore, immune markers related to IFN signaling were upregulated. Future studies should be directed at unraveling the precise physiological consequences of these changes.

## Conflict of Interest

None declared.

## Supporting Information

**Table S1.** Expression of adaptive and innate immune markers in muscle biopsies before and after 10 days of cold acclimation in healthy subjects.
