# Supplementary material for: Cold acclimation affects immune composition in skeletal muscle of healthy lean subjects
Source: Physiol Rep. 2015 Jul 6;3(7):e12394. doi: 10.14814/phy2.12394 (PMC4552515; doi:10.14814/phy2.12394)
Supplement: Supplementary file 1 [file phy20003-e12394-sd1.docx]

**Suppl Table 1. Expression of adaptive and innate immune markers in muscle biopsies before and after 10 days of cold acclimation in healthy subjects**

| **Gene** | **Baseline** | **Cold (fold change *vs* baseline)** | **P-value** |
| --- | --- | --- | --- |
| ***Immune cell subset markers*** | | | |
| BLR1 (B-cells) | 1.00 ± 0.07 | 0.89 ± 0.04 | 0.16 |
| CD19 (B-cells) | n.d. | n.d. | n.a. |
| NCAM1 (NK cells) | 1.00 ± 0.16 | 1.04 ± 0.19 | 0.87 |
| ***T cell subsets*** | | | |
| CD3E | 1.00 ± 0.12 | 1.54 ± 0.59 | 0.38 |
| CD4 | 1.00 ± 0.05 | 1.12 ± 0.09 | **0.076** |
| CD8A^#^ | 1.00 ± 0.16 | 0.85 ± 0.08 | 0.19 |
| CCR7 | 1.00 ± 0.08 | 0.83 ± 0.09 | 0.20 |
| PTPRCv1 | n.d. | n.d. | n.a. |
| PTPRCv2 | 1.00 ± 0.15 | 1.11 ± 0.25 | 0.33 |
| AIRE | 1.00 ± 0.14 | 0.87 ± 0.10 | 0.55 |
| ***Th1 response*** | | | |
| CXCL10 | 1.00 ± 0.10 | 1.04 ± 0.10 | 0.74 |
| IFNG | 1.00 ± 0.29 | 0.47 ± 0.09 | 0.12 |
| IL1B | 1.00 ± 0.12 | 0.96 ± 0.10 | 0.79 |
| IL2 | 1.00 ± 0.09 | 0.92 ± 0.07 | 0.48 |
| IL15 | 1.00 ± 0.22 | 0.99 ± 0.13 | 0.95 |
| TBX21 | n.d. | n.d. | n.a. |
| TNF | 1.00 ± 0.07 | 0.87 ± 0.03 | 0.13 |
| ***Th2 response*** | | | |
| GATA3 | 1.00 ± 0.17 | 1.12 ± 0.40 | 0.78 |
| IL4 | n.d. | n.d. | n.a. |
| IL4δ2^#^ | 1.00 ± 0.59 | 0.79 ± 0.37 | 0.76 |
| IL5 | n.d. | n.d. | n.a. |
| IL6 | 1.00 ± 0.09 | 0.87 ± 0.05 | 0.17 |
| IL9 | 1.00 ± 0.09 | 1.06 ± 0.11 | 0.66 |
| IL13 | 1.00 ± 0.15 | 0.85 ± 0.14 | 0.36 |
| ***Th17 response*** | | | |
| IL17A^#^ | 1.00 ± 0.22 | 0.79 ± 0.00 | 0.33 |
| RORC | **1.00 ± 0.06** | **0.72 ± 0.06** | **0.0038*** |
| NEDD4L | **1.00 ± 0.05** | **0.85 ± 0.03** | **0.04*** |
| IL22RA1^#^ | 1.00 ± 0.00 | 1.13 ± 0.08 | 0.12 |
| ***Treg markers*** | | | |
| CTLA4 | 1.00 ± 0.65 | 0.53 ± 0.15 | 0.49 |
| FOXP3 | **1.00 ± 0.06** | **0.87 ± 0.04** | **0.043*** |
| IL10 | 1.00 ± 0.45 | 1.14 ± 0.34 | 0.81 |
| IL2RA^#^ | 1.00 ± 0.16 | 1.15 ± 0.30 | 0.50 |
| IL7R | 1.00 ± 0.13 | 0.72 ± 0.05 | **0.08** |
| LAG3 | 1.00 ± 0.13 | 0.75 ± 0.15 | 0.12 |
| TGFB1 | 1.00 ± 0.05 | 1.06 ± 0.03 | 0.18 |
| TNFRSF18 | 1.00 ± 0.05 | 0.94 ± 0.06 | 0.47 |
| ***Cytotoxicity markers*** | | | |
| GNLY | 1.00 ± 0.17 | 1.26 ± 0.30 | 0.41 |
| GZMA | 1.00 ± 0.06 | 0.91 ± 0.09 | 0.36 |
| GZMB | 1.00 ± 0.10 | 1.08 ± 0.08 | 0.52 |
| PRF1 | 1.00 ± 0.08 | 0.93 ± 0.07 | 0.56 |
| ***Anti-microbial activity*** | | | |
| LTF | 1.00 ± 0.09 | 0.93 ± 0.12 | 0.46 |
| ***Macrophage markers*** | | | |
| CD14 | 1.00 ± 0.11 | 0.95 ± 0.08 | 0.61 |
| CD163 | 1.00 ± 0.23 | 0.90 ± 0.11 | 0.62 |
| CD209 | **1.00 ± 0.05** | **0.85 ± 0.03** | **0.04*** |
| CCL2 | 1.00 ± 0.16 | 1.11 ± 0.28 | 0.75 |
| CCL3 | 1.00 ± 0.18 | 0.81 ± 0.06 | 0.35 |
| CCL4 | 1.00 ± 0.06 | 0.95 ± 0.05 | 0.43 |
| CCL5 | 1.00 ± 0.05 | 0.91 ± 0.06 | 0.33 |
| CCL22 | **1.00 ± 0.17** | **0.50 ± 0.12** | **0.038*** |
| CXCL13 | **1.00 ± 0.07** | **0.83 ± 0.05** | **0.049*** |
| IL12A | 1.00 ± 0.23 | 1.15 ± 0.39 | 0.75 |
| IL12B^#^ | **1.00 ± 0.00** | **2.41 ± 0.60** | **0.029*** |
| IL23A | 1.00 ± 0.20 | 1.04 ± 0.16 | 0.86 |
| ***Scavenger receptors*** | | | |
| MARCO | 1.00 ± 0.23 | 2.78 ± 1.46 | 0.23 |
| ***Pattern recognition receptors*** | | | |
| CLEC7A | 1.00 ± 0.30 | 0.99 ± 0.33 | 0.99 |
| MRC1 | 1.00 ± 0.25 | 1.17 ± 0.25 | 0.65 |
| MRC2 | 1.00 ± 0.06 | 1.08 ± 0.08 | 0.36 |
| NOD1 | 1.00 ± 0.06 | 0.82 ± 0.07 | **0.085** |
| NOD2 | 1.00 ± 0.08 | 0.83 ± 0.07 | **0.068** |
| TLR1^#^ | **1.00 ± 0.00** | **2.01 ± 0.48** | **0.04*** |
| TLR2 | 1.00 ± 0.10 | 0.88 ± 0.08 | 0.37 |
| TLR3 | 1.00 ± 0.07 | 0.97 ± 0.06 | 0.75 |
| TLR4 | 1.00 ± 0.17 | 1.15 ± 0.15 | 0.57 |
| TLR5 | 1.00 ± 0.36 | 0.90 ± 0.31 | 0.75 |
| TLR6^#^ | 1.00 ± 0.22 | 0.83 ± 0.15 | 0.55 |
| TLR7 | 1.00 ± 0.07 | 0.98 ± 0.05 | 0.82 |
| TLR8 | n.d. | n.d. | n.a. |
| TLR9 | 1.00 ± 0.05 | 0.89 ± 0.03 | 0.14 |
| TLR10 | n.d. | n.d. | n.a. |
| ***Inflammasome components*** | | | |
| NLRC4 | 1.00 ± 0.04 | 0.89 ± 0.05 | 0.13 |
| NLRP1 | 1.00 ± 0.24 | 0.82 ± 0.19 | 0.56 |
| NLRP2 | 1.00 ± 0.06 | 0.89 ± 0.04 | 0.16 |
| NLRP3 | 1.00 ± 0.33 | 1.55 ± 0.41 | 0.35 |
| NLRP4 | 1.00 ± 0.09 | 0.92 ± 0.08 | 0.62 |
| NLRP6 | n.d. | n.d. | n.a. |
| NLRP7 | n.d. | n.d. | n.a. |
| NLRP10 | n.d. | n.d. | n.a. |
| NLRP11 | n.d. | n.d. | n.a. |
| NLRP12 | n.d. | n.d. | n.a. |
| NLRP13^#^ | 1.00 ± 0.25 | 0.93 ± 0.20 | 0.83 |
| ***IFN signaling genes*** | | | |
| CD274 | **1.00 ± 0.14** | **1.36 ± 0.14** | **0.049*** |
| FCGR1A | 1.00 ± 0.05 | 1.12 ± 0.08 | 0.12 |
| GBP1 | 1.00 ± 0.12 | 1.15 ± 0.09 | 0.33 |
| GBP2 | 1.00 ± 0.06 | 0.91 ± 0.06 | 0.22 |
| GBP5 | n.d. | n.d. | n.a. |
| IFI6 | 1.00 ± 0.12 | 0.84 ± 0.03 | 0.25 |
| IFI16 | 1.00 ± 0.11 | 0.81 ± 0.07 | 0.13 |
| IFI35^#^ | 1.00 ± 0.87 | 0.11 ± 0.0 | 0.31 |
| IFI44 | 1.00 ± 0.05 | 1.31 ± 0.25 | 0.22 |
| IFI44L | 1.00 ± 0.10 | 0.79 ± 0.10 | **0.08** |
| IFIH1 | 1.00 ± 0.35 | 1.08 ± 0.31 | 0.84 |
| IFIT2 | 1.00 ± 0.10 | 1.06 ± 0.11 | 0.7 |
| IFIT3 | 1.00 ± 0.07 | 0.89 ± 0.04 | 0.11 |
| IFIT5^#^ | 1.00 ± 0.14 | 0.92 ± 0.07 | 0.59 |
| IFITM1/3 | **1.00 ± 0.05** | **1.11 ± 0.07** | **0.026*** |
| INDO | 1.00 ± 0.11 | 0.90 ± 0.09 | 0.3 |
| IRF7 | n.d. | n.d. | n.a. |
| OAS1^#^ | 1.00 ± 0.71 | 3.18 ± 1.52 | **0.098** |
| OAS2 | 1.00 ± 0.10 | 1.09 ± 0.10 | 0.51 |
| OAS3 | 1.00 ± 0.11 | 1.15 ± 0.09 | 0.4 |
| SOCS1 | 1.00 ± 0.13 | 0.90 ± 0.06 | 0.52 |
| STAT1 | 1.00 ± 0.02 | 0.97 ± 0.05 | 0.39 |
| STAT2 | **1.00 ± 0.04** | **1.10 ± 0.04** | **0.028*** |
| TAP1 | **1.00 ± 0.03** | **1.12 ± 0.05** | **0.0065*** |
| TAP2 | 1.00 ± 0.08 | 0.94 ± 0.06 | 0.55 |
| ***Apoptosis / Survival*** | | | |
| CASP8 | 1.00 ± 0.13 | 1.32 ± 0.11 | **0.054** |
| BCL2 | **1.00 ± 0.08** | **0.83 ± 0.06** | **0.042*** |
| FASLG | n.d. | n.d. | n.a. |
| FLCN1 | **1.00 ± 0.05** | **0.85 ± 0.06** | **0.048*** |
| TNFRSF1A | 1.00 ± 0.04 | 1.12 ± 0.09 | 0.14 |
| TNFRSF1B | n.d. | n.d. | n.a. |
| ***Small GTPases / (Rho)GTPase activating proteins*** | | | |
| ASAP1 | 1.00 ± 0.80 | 1.67 ± 1.11 | 0.63 |
| RAB13 | 1.00 ± 0.06 | 1.15 ± 0.11 | 0.12 |
| RAB24 | 1.00 ± 0.07 | 1.05 ± 0.04 | 0.41 |
| RAB33A^#^ | 1.00 ± 0.32 | 0.36 ± 0.12 | **0.08** |
| TAGAP | 1.00 ± 0.22 | 0.70 ± 0.14 | **0.088** |
| TBC1D7 | n.d. | n.d. | n.a. |
| ***Chemokines*** | | | |
| CCL11^#^ | 1.00 ± 0.22 | 1.54 ± 0.39 | 0.18 |
| CCL13 | n.d. | n.d. | n.a. |
| CCL19 | 1.00 ± 0.09 | 1.01 ± 0.09 | 0.92 |
| CXCL9 | 1.00 ± 0.22 | 1.03 ± 0.12 | 0.92 |
| CX3CL1 | 1.00 ± 0.16 | 1.01 ± 0.17 | 0.95 |
| ***Cell growth / proliferation*** | | | |
| BMP6 | 1.00 ± 0.18 | 0.76 ± 0.08 | 0.17 |
| TGFBR2 | 1.00 ± 0.06 | 1.06 ± 0.05 | 0.39 |
| AREG | n.d. | n.d. | n.a. |
| EGF | 1.00 ± 0.13 | 0.96 ± 0.10 | 0.69 |
| VEGF | 1.00 ± 0.10 | 0.91 ± 0.08 | 1.0 |
| ***Cell activation*** | | | |
| HCK | 1.00 ± 0.19 | 0.80 ± 0.06 | 0.27 |
| LYN | 1.00 ± 0.09 | 0.91 ± 0.05 | 0.43 |
| SLAMF7 | 1.00 ± 0.23 | 0.79 ± 0.07 | 0.41 |
| ***Transcriptional regulators / activators*** | | | |
| CAMTA1 | n.d. | n.d. | n.a. |
| TWIST1 | n.d. | n.d. | n.a. |
| ZNF331 | 1.00 ± 0.19 | 0.72 ± 0.14 | **0.07** |
| ZNF532 | 1.00 ± 0.07 | 0.89 ± 0.04 | 0.15 |
| ***Intracellular transport*** | | | |
| SEC14L1 | 1.00 ± 0.04 | 0.94 ± 0.04 | 0.11 |
| KIF1B | 1.00 ± 0.08 | 0.92 ± 0.08 | 0.35 |
| ***Inflammation*** | | | |
| DSE | 1.00 ± 0.16 | 0.88 ± 0.08 | 0.43 |
| MMP9 | 1.00 ± 0.07 | 1.01 ± 0.09 | 0.91 |
| SPP1 | n.d. | n.d. | n.a. |
| TIMP2 | 1.00 ± 0.05 | 1.03 ± 0.07 | 0.64 |
| TNIP1 | 1.00 ± 0.10 | 0.91 ± 0.08 | 0.26 |
| ***Mitochondrial Stress / Proteasome*** | | | |
| HPRT | n.d | n.d. | n.a. |

Data are presented as mean ± SEM and expressed relative to baseline. *P<0.05 *vs.* before cold acclimation. ^#^ Low expression of gene. n.d., not detectable; n.a., not applicable
